# Supplementary material for: Are carbon emissions trading and green financial instruments synergistic? -Comprehensive quantitative research based on content analysis
Source: PLoS One. 2024 Mar 7;19(3):e0298601. doi: 10.1371/journal.pone.0298601 (PMC10919601; doi:10.1371/journal.pone.0298601)
Supplement: S1 Data — (ZIP) [file pone.0298601.s001.zip › Data/地级市Z_F工作报告.zip!/╔╜╬≈/┴┘╖┌╩╨/└┤╫╘╣1⁄2╓┌║┼í╛┬φ┐╦╩2╛▌═°í┐_2017.docx]

各位代表：

　　现在，我代表市人民政府向大会报告工作，请予审议，并请市政协委员和其他列席人员提出意见。

　　一、2016年工作回顾

　　刚刚过去的一年，面对经济下行的较大压力，面对去产能调结构的艰巨任务，面对大气污染的严峻形势和不利影响，在省委、省政府和市委的坚强领导下，在市人大、市政协的监督支持下，市政府深入贯彻落实党的十八大和十八届三中、四中、五中、六中全会精神和习近平总书记系列重要讲话精神，按照省委“一个指引、两手硬”重大思路和要求，围绕市委、市政府“345”发展战略和“12345”工作思路，坚持以新发展理念为引领，牢牢把握稳中求进工作总基调，深入推进供给侧结构性改革，全市经济社会发展实现稳中向好、稳中有进。

　　——凝心聚力稳增长，应对经济下行取得明显成效。坚持超前研判、精准施策、积极作为，在全省率先开展项目开工“百日攻坚”行动、“各类项目受理大起底”活动，扎实推动千名干部入企服务，有效顶住了经济下行压力，主要指标实现下半年好于上半年目标。全市地区生产总值完成1205.2亿元，同比增长3.4%，增速比上半年提高1.6个百分点;规模以上工业增加值完成309.7亿元，下降0.4%，比上半年增速提高0.2个百分点;一般公共预算收入完成86亿元，下降2.5%，比上半年增速提高1.6个百分点;固定资产投资完成1394.3亿元，其中5000万元以上项目投资比重比上半年提高4.6个百分点;社会消费品零售总额完成609.4亿元，增长6.5%，比上半年增速提高0.6个百分点;城镇居民人均可支配收入完成27085元，增长6.2%;农村居民人均可支配收入完成10005元，增长6.7%，城乡居民收入增速均高于全省平均水平。

　　——坚定不移调结构，产业转型升级实现稳中有进。持之以恒优化产业布局，强化产业培育，全市三次产业比重优化为8:46.5:45.5。第三产业占比提高1.8个百分点，对经济增长的支撑作用显著增强。其中旅游总收入达到375亿元，同比增长27.1%，对第三产业发展的贡献率达到84.1%。工业方面，传统产业仍然是主力军，但全年新兴产业投资达到工业领域总投资的72.3%，新兴产业增速、贡献率双超传统产业，非煤产业贡献率超过煤炭产业，实现了动力转换。特别是沃特玛新能源汽车等现代装备制造业快速发展，比上年增长31.5%。整个工业经济，价格得到提升，结构得到优化，有了长足发展。农业生产稳中调优，粮食总产53.6亿斤，同比增长13.4%，秋粮产量创历史新高。“四个百万亩”规模不断壮大，新增设施蔬菜1.18万亩、水果11.6万亩、干果10万亩、中药材13.6万亩。肉、蛋、奶总产量分别达32.4万吨、17.2万吨、7.5万吨。

　　——毫不动摇促改革，发展活力和内生动力不断增强。扎实推进“三去一降一补”，压减炼铁产能82万吨、煤炭产能270万吨、退出煤矿矿井5座，在原煤产量同比下降7.2%、生铁下降9%、粗钢下降6.7%的同时,工业效益实现总体回升;商品房去库存成效明显，消化周期同比缩短4.1个月;结构性减税降费政策全面落实，工业企业每百元主营业务收入成本同比下降3.95元。财政一般公共预算收入质量显著改善，税收收入占比超过非税收入，由2013年的45.8%提高到2016年的58.41%，提高了12.61个百分点。金融创新、银企合作效果良好，银行各项贷款余额达到1184亿元，同比增长9.7%，有力地支持了实体经济发展。促进企业与银行达成30亿元存量债务置换贷款，年可为企业节约利息1亿多元。融资渠道不断拓宽，融资手段更为丰富，“新三板”挂牌企业达到5家，后备企业达到30多家，在省股权交易中心挂牌的企业达到170余家，初步形成了“梯次推进、持续发展”的良好格局。不等不靠推改革、破难题，政务服务国家级标准化试点在全省率先启动，“放管服效”改革纵深推进，“营改增”全面推开，“五证合一”全面实施，开发区改革创新加快推进。承接、取消、下放、调整行政审批事项109项。科技体制改革不断深化，“双创”氛围日趋浓厚。建成省级创业基地6个、市级创业基地1个、小微企业服务站30个，培育“小升规”企业24户，创办小微企业3592户。全市医疗卫生体制、国有林场改革、集体林权配套制度、农村土地确权等各项改革都取得积极成效。加快构建开放型经济新体制，积极承接发达地区产业转移，签约中美合资JDH、合肥华翔汽车件等项目151个，签约资金2514亿元，新设外商投资企业4家，合同利用外资总额1.9亿美元，同比大幅增长。

　　——坚持不懈优环境，城乡面貌发生积极变化。坚持从一点一滴着手，从功能特色着力，完善城镇功能与建设美丽乡村双轮驱动，促进人口、产业聚集，全市常住人口城镇化率达到50.03%，城镇人口首次超过乡村人口。侯马市成功获批国家第三批新型城镇化试点城市。全面加强城乡基础设施建设。市区完成鼓楼南北街、燕尔巷、东关步行街等拓宽改造工程，有效缓解了老城区交通拥堵状况;加快城市供热、供气、供水管网等市政基础设施建设，完成老百汇搬迁，坚持“冬病夏治”，组织开展露天烧烤、建筑施工扬尘、重点区域环境和秸秆禁烧4个专项整治行动，改善了市容环境，提升了城市形象。加快城乡交通基础设施建设，完成投资63.6亿元，吉河高速全面通车，长临高速、霍永高速西段二期工程加快推进，新改建国省干线公路41公里、农村公路915公里。超额完成农村人居环境改善“四大工程”年度任务，创建省级美丽宜居示范村13个，创建清洁达标村1330个。

　　——倾心尽力惠民生，群众获得感更实更足。坚持问题导向和需求导向，着眼公平普惠、成果共享，全力做好群众最关心、最急需的民生实事。优先增加民生投入，全年民生支出261.5亿元，同比增长7.5%，占一般公共预算支出的83.7%。全面打响脱贫攻坚战，精准扶贫脱贫基础工作进一步夯实，扶贫资金整合到位，产业扶贫成效明显，易地扶贫搬迁有序推进，教育扶贫应助尽助，健康扶贫政策到位，生态扶贫、金融扶贫、电商扶贫、旅游扶贫成效初显，光伏扶贫、消费扶贫、中证扶贫板特色鲜明，共计182个贫困村摘帽、81856人脱贫，超额完成省市下达的目标任务，实现了脱贫攻坚开局之年首战首捷。加快发展文化教育事业，市图书馆完成主体工程，市奥体中心体育场、市文化中心加快推进。79件作品获“山西广播电视奖”。新建农村幼儿园24所。乡宁、永和、大宁、隰县、蒲县5县通过义务教育均衡县认定。全市高考达二本B类以上16535人，达线率42.9%。山西师大新校区、师大临汾学院搬迁工程取得积极进展。不断提升卫计水平，城市公立医院改革前期工作准备就绪;市区医疗资源整合全面完成;在全省率先开展了“全民健康·临汾在行动”活动;侯马市入选我省唯一的全国健康城市试点市。全面加强社会保障，多渠道拓宽就业岗位，城镇新增就业51258人。企业和机关事业单位退休人员养老金待遇进一步提高，落实了乡镇补贴及职务与职级并行制度，提高了城乡低保标准。建成各类保障性住房9768套，完成农村危房改造11180户。11个县27个乡镇采煤沉陷区治理工程全面开工。加大生态保护力度，初步建成吕梁山生态林业示范区、民生林业样板区，全年完成营造林46.19万亩，超省定任务0.9个百分点。实施国家节能减排财政政策综合示范城市项目31个。超额完成黄标车及老旧车淘汰任务。市区投放公共自行车5055辆。全市新增更新纯电动公交车732辆，其中市区新增更新200辆，成为全省首个纯电动公交车全覆盖城市。持续加强安全工作。强化安全责任，加强安全监管，深入开展安全风险隐患大排查大整治，安全生产形势持续稳定好转。深入推进“平安临汾”“法治临汾”建设，全面启动“七五”普法规划。连续三年实施“六大惠民工程”，人民群众得到了更多实惠。

　　——狠抓落实提效能，政府自身建设取得新的进展。坚持依法行政，自觉接受人大工作监督、法律监督和政协民主监督，办理人大代表建议172件、市政协提案234件。深入开展“两学一做”学习教育，严格落实从严治党主体责任，有力营造了树正气、讲团结、聚合力、促转型的浓厚氛围。坚持全面提升干部素质，组织系列讲座10余期。认真落实“13710”工作制度，建立了市政府双月县长例会制度和工作周报制度，行政效能进一步提升。

　　与此同时，国防动员、双拥共建和优抚安置工作进一步加强。民族宗教、外事侨务、群众文化体育、老龄、残疾人、红十字会、妇女儿童等事业都有了新进步，人防、统计、食药监、工商、质监、审计、文物、地方志、气象、地震、档案、住房公积金等工作均取得新进展。

　　各位代表，一年来，我们经受了多重困难挑战的考验，进一步强化了新发展理念，坚定了转型综改、创新驱动、绿色发展的信心和决心。困难中的转机来之不易，压力下的收获弥足珍贵。这是省委、省政府和市委正确领导的结果，是市人大、市政协大力支持的结果，是全市上下万众一心、众志成城、团结奋斗的结果。在此，我代表市政府，向全市人民，向人大代表、政协委员，各民主党派、工商联、无党派人士和人民团体，向驻临部队、武警官兵和所有关心支持临汾发展的各界朋友，表示崇高的敬意和衷心的感谢!各位代表，在总结成绩的同时，我们也清醒地认识到，当前，我市发展面临的困难更多，挑战更大，形势更为严峻。特别是生态环境问题已经成为经济社会发展的首要问题，到了一个非解决不可的地步。受不利气候条件和我市特殊的地理地形、产业结构、传统燃煤取暖方式的影响，受重视程度不够、监督管理不力、应对经验不足的影响，2016年我市空气质量六项监测指标均不降反升，重度及以上污染天数同比增加31天，优良天数同比减少22天。尤其是二氧化硫浓度超标问题突出，受到媒体和社会的普遍关注，被环保部约谈，并暂停新增大气污染物排放项目的环评审批。去冬以来，共启动重污染天气预警19次，其中红色预警6次，造成大范围、长时间限产停产，对经济发展、居民收入、生产生活造成了一定影响，我们深感痛心和内疚。不解决好这个问题，临汾就没有出路;不解决好这个问题，我们就会有负于全市人民的重托。

　　与此同时，经济社会发展存在诸多短板。全市经济仍然处在最为困难的时期，多年积累的“一煤独大”结构性矛盾远未从根本上解决，企稳向好的基础还不牢固，特别是经济增长还缺乏大项目支撑、新要素新动能支撑和强有力的人才支撑;企业负债率偏高、财政收支不平衡、居民增收困难等问题仍然突出;城乡发展不平衡，脱贫攻坚难度大，民生社会事业欠账较多，补短板任务十分艰巨;安全生产隐患仍然较多，社会管理粗放，基层社会治理能力和水平有待提高;政府职能转变还不到位，少数干部法治意识、责任意识、服务意识还不强，存在部署落实不到位、执行政策不严格等情况。对于这些困难和问题，我们要以对党和人民高度负责的态度，不回避、不懈怠，采取更积极、更有效的措施，切实加以解决。

　　二、2017年目标任务和重点工作

　　今年是实施“十三五”规划的重要一年，是推进供给侧结构性改革和转型综改的深化之年，是我市走出经济和环保困难局面的攻坚之年。政府工作的总体要求是：深入贯彻习近平总书记系列重要讲话精神和治国理政新理念新思想新战略，统筹落实“五位一体”总体布局和“四个全面”战略布局，按照省委“一个指引、两手硬”的重大思路和要求，紧紧围绕市委、市政府“345”发展战略和“12345”工作思路，坚持新发展理念，坚持稳中求进工作总基调，坚持深化供给侧结构性改革与深化转型综改区建设有机结合，坚持以提高发展质量和效益为中心，全面推进转型综改、创新驱动，持续加强生态环境治理，坚定走加快转型、绿色发展之路，为决胜全面小康、实现振兴崛起奠定更加坚实的基础，以优异成绩迎接党的十九大胜利召开。

　　主要预期指标是：地区生产总值增长5.5%;规模以上工业增加值增长3%;社会消费品零售总额增长7%;一般公共预算收入力争完成86亿元，与上年持平;城镇居民人均可支配收入增长6%，农村居民人均可支配收入增长6.5%;居民消费价格涨幅控制在3%左右;城镇新增就业4.5万人，城镇登记失业率控制在4.2%以内。固定资产投资完成省定任务。

　　约束性指标是：资源节约方面，万元地区生产总值能耗下降4.219%、二氧化碳排放量下降3.9%、用水量下降2.8%。环境质量方面，细颗粒物(PM2.5)浓度下降比例、空气质量优良天数比例、达到或好于Ⅲ类水体比例、劣V类水体比例完成省下达任务;二氧化硫、化学需氧量、氨氮、氮氧化物减排幅度，完成财政部和国家发改委下达任务。民生改善方面，农村贫困人口脱贫7.4万人，城镇棚户区住房改造数量完成省定任务。

　　各位代表，完成上述目标，我们将着重把握稳与进、质与量、供与需、政与企、生态文明建设与经济社会发展等方面的关系，更加突出质量效益中心，更加突出创新驱动引领，更加突出绿色发展方向，更加突出以民为本理念，更加突出打基础利长远导向，以“功成不必在我”的境界，把主要精力集中到夯实发展基础、补齐发展短板、打牢发展根基上来，坚决不要带血的GDP、不要污染环境破坏生态的GDP、不要掺假带水分的GDP，努力创造有质量有效益真金白银的GDP、绿水青山可持续发展的GDP、老百姓有实实在在获得感和幸福感的GDP!

　　今年，重点抓好以下工作：

　　(一)坚定不移保持经济平稳健康发展。着力巩固“稳”的基础，积蓄“进”的力量，不断扩大向好态势，为长远发展谋势蓄力。

　　以项目建设为支撑精准扩大有效投资。牢牢把握国家投资方向和重点，围绕产业转型升级、重大基础设施、城乡一体化、社会事业等，实施645个有重大引领支撑作用的重点项目，总投资3935亿元，当年计划完成投资674亿元。创新投资机制和方式，大力推广政府和社会资本合作模式，对已进入重大项目库的PPP项目，要加大实施力度。深化投资体制改革，推动投资审批制度规范化、标准化，进一步健全完善领导包联、定期调度、双向协调等工作推进机制，最大限度减少核准事项和前置条件，探索建立多评合一、多审合一、多图联审、联合验收等新模式。继续深入开展“引老乡、回故乡、建家乡”活动，积极承接产业转移，推进中美合资JDH、侯马开发区宏凯集团电子类产品综合产业基地等项目建设，精准引进大项目、好项目。要坚决防止造成新的产能过剩、重复建设、库存增加、杠杆扩大的投资。

　　以促进金融业发展为手段全力振兴实体经济。金融业发展不足已经成为制约我市实体经济健康发展的重要因素。要大力推进金融业创新发展，做好我市首家民营银行黄河银行筹建和村镇银行普设工作。逐步推进县级农信社改制为农村商业银行。鼓励尧都农商银行发起设立消费金融公司。充分争取和利用国家政策性贷款及险资入临政策，加强政银企合作平台建设，推动重点项目融资。加快多层次资本市场体系建设，大力发展股权投资基金、创业投资基金等，引导民间资本有序进入实体经济。加强诚信体系建设，提升守信意识，打击违约行为，加强信用评级。有效防范地方性金融机构风险，遏制地方法人金融机构不良贷款反弹和资产质量恶化。严厉打击非法集资，确保区域金融稳定。

　　以做大做强为目标大力促进民营经济发展。全面落实《关于进一步支持民营经济健康发展的措施》，扎实开展送政策、送专家、送服务“三送”活动。重点抓好尧都、侯马两个省级中小企业“双创示范县”工作，培育洪洞中小企业创业基地和尧都、侯马电商物流创业基地;新创办小微企业3000户，力争完成10家企业股份制改造，争取更多的企业在“新三板”挂牌。进一步优化民营经济发展环境，依法加强对各种所有制法人产权保护，保证民营经济依法平等使用生产要素、公平参与市场竞争、同等受到法律保护。加快构建“亲”“清”新型政商关系，以“亲”的情感服务民营经济，以“清”的原则规范权力行为，进一步营造尊重创新创造、尊崇企业家精神的浓厚氛围，让企业家在振兴全市经济中创造新业绩、铸就新辉煌。

　　(二)坚定不移加快新旧动能转换。目前，我市经济正处于新旧动能转换的艰难进程中，传统产业仍是主力军、主税源，新兴产业则是新动力、新支撑，二者犹如我市经济发展之两翼。要大力实施工业振兴计划，逐步形成“4+6+N”现代工业产业体系，大力实施科技创新，大力发展现代服务业，让传统动能焕发生机，让新动能尽快挑起大梁。

　　加快改造传统产业。突出主焦煤品牌，积极筹建临汾焦煤集团，增加我市在国内主焦煤市场上的话语权。全年竣工投产矿井8座，产能660万吨/年;现代化矿井不少于10座，一级安全质量标准化矿井不低于20座，其余全部达到二级标准。大力推进新一轮技术改造和设备更新，重点实施产品结构调整、装备水平提升、产业整合重组“三大工程”，抓好曲沃立恒精炼炉、安泽永鑫甲醇制丙烯、侯马北铜铜精矿综合回收等项目建设。

　　加快发展新兴产业。围绕现代煤化工、现代装备制造、新能源、新材料、新医药、节能环保等六大领域，重点抓好沃特玛新能源汽车产业园、霍州亿能电器新能源汽车、西山煤层气开发以及永和天然气勘探开发、汾西其亚240万吨氢氧化铝等项目建设，促进产业集群发展，争取新兴产业占比提高2个百分点左右。积极发展大数据、云计算、物联网等前沿产业，建设华为(临汾)云计算大数据产业园，启动临汾(尧庙)“互联网+”智慧产业园项目。

　　加快提升创新动力。抓好“双创”系列政策落地，建设一批“互联网+”新型孵化器，力争年内培育市级众创空间3-4家、“星创天地”2-3个。加大高新技术企业培育力度，引导和支持创新要素向企业集聚，鼓励企业不断提高研发水平，支持企业开发具有核心竞争力、高附加值和自主知识产权的产品，力争全年新建1家工程技术研究中心、培育1家省级重点实验室、新认定4家高新技术企业。加快补齐人才短板，完善本土科技人才激励机制，持续用好“市长创新奖”，吸引更多创新型人才落户临汾。积极与山西师大、山西中医学院等高校开展“市校合作”，实现产、学、研无缝对接。

　　加快发展现代服务业。推进“互联网+物流”发展，加快建设山西方略洋货码头、晋润农副产品冷链物流、兴荣物流园区等项目;推进电子商务提速发展，着力建设沿汾电商发展带和淘宝、京东、苏宁特色中国临汾馆，实现“网货下乡”和“农产品进城”;推进传统服务业提质发展，突出业态创新、管理创新和服务创新，实现餐饮、家政、房地产、健康、养老等服务便利化、精细化、品质化。

　　(三)坚定不移发展文化旅游产业。围绕把文化旅游业打造成为我市战略性支柱产业的目标，突出“中国根·黄河魂”品牌建设，着力破解体制机制障碍，加大旅游景区景点建设力度，促进文化与旅游深度融合，共赢发展。

　　加快旅游体制机制改革。在成立市旅游委的基础上，在尧都、洪洞、吉县、霍州等重点县(市、区)设立旅游发展委员会。组建市文化旅游产业集团，全面整合我市文化旅游资源。制定《临汾市旅游资源开发保护管理办法》和《临汾市旅游业发展提升规划》，进一步理顺旅游景区管理体制、经营机制和各类利益关系，引进一批战略合作者，培育一批经营主体，6月底前完成10个景区、景点体制机制改革。牢固树立大规划引领、市场化运作理念，持续构建政府引导、社会参与的旅游资源发展模式，实现旅游景区所有权、经营权分离。

　　联动开发文化旅游资源。按照“旅游+”的思路，把握“安、顺、诚、特、需、愉”六字要诀，大力发展全域旅游。县(市、区)联动，全力推进大槐树、壶口、云丘山、人祖山、丁村、广胜寺、晋国博物馆等5A、4A级景区创建工作，加快推进陶寺遗址博物馆、陶寺遗址展示中心、尧陵以及云丘山、仙洞沟、乾坤湾等景区建设，同步推进停车场、旅游厕所、标识牌等基础配套服务设施建设。加快旅游公路建设，力争开工210公里，完成150公里。

　　促进文化与旅游深度融合。以平阳记忆和印象临汾为主线，实施戏曲传承发展振兴工程、《赵城金藏》复制工程、非物质文化遗产推广工程、文艺精品创作工程、品牌文体活动打造工程、舆论宣传引导工程“六大工程”，唤起城市记忆，彰显文化魅力，推动文化与旅游融合发展。

　　(四)坚定不移全面深化改革开放。始终坚持改革不停顿、开放不止步，以供给侧结构性改革、综改试验区建设为契机，更好发挥改革的牵引作用、开放的先导作用，不断增强经济社会发展的动力与活力。

　　深入推进供给侧结构性改革。“三去一降一补”是供给侧结构性改革的重要任务，必须巩固成果，继续深化，确保取得新的实质性进展。更多运用市场化、法治化方式，坚决完成淘汰落后产能任务，依法依规处置“僵尸企业”;综合采取提高棚户区和城中村改造货币化安置率、鼓励补贴农民进城购买新建商品房等措施，有效化解房地产库存，促进房地产平稳健康发展;充分运用股权融资、市场化债转股等多种手段，提高直接融资比重，积极稳妥降低企业杠杆率，控制和化解政府融资风险。继续打好降低制度性交易成本、生产要素成本等“组合拳”，切实降低企业成本;加大公共服务、基础设施、生态环保、防灾减灾等领域补短板力度。

　　深入推进综改试验区建设。把深化转型综改试验区建设与深化供给侧结构性改革有机结合起来，通过推进一批重大改革、重大事项、重大项目和重大课题，破解体制机制难题，使改革红利尽快转化为发展动力。加大我市转型综改示范区建设申报力度，重点在产业转型、环境治理、主体功能区建设、行政区划调整等领域展开探索，努力将百里汾河生态文明保护区建设成国家级生态文明试验区，将百里汾河生态经济带打造成产城融合发展示范区，推动综改试验区建设取得新突破。

　　深入推进开发区体制机制改革。科学编制开发区总体发展规划，将经济社会发展规划、城乡建设规划、土地利用规划、产业发展规划、环境保护规划合并为“一本规划、一张蓝图”。理顺开发区管理体制、运行机制和各类利益关系，建立专业化、市场化、国际化的管理运行机制，实行领导班子任期制、全员岗位聘任制和绩效工资制。实行开发区改革创新发展定期协调会议制度。今年重点推动临汾、侯马开发区以及侯马综合保税区、洪洞经济开发区、襄汾经济开发区、尧都高新技术开发区扩区升级、申报工作，确保开发区改革创新工作走在全省前列。

　　深入推进国资国企改革。按照“创新发展一批，重组整合一批，清理退出一批”的思路，加快国有企业公司化改革，加快发展混合所有制经济，优化国有资本结构，加快“僵尸”企业退出市场步伐。探索组建国有资本投资(运营)公司，以管资本为主加强国有资产监管，实现国有资产监管全覆盖。

　　深入推进重点领域关键性改革。深化“放管服效”改革，进一步完善政府权力清单和责任清单，加快推行负面清单制度。完善事中事后监管，实现“双随机、一公开”监管全覆盖。推进综合执法改革，运用智能监管手段，提高监管效能。加快实行“多证合一”“一照多址”等便利化措施，推进企业登记全程电子化。深化价格机制改革，最大限度减少政府定价项目、降低收费标准。统筹抓好电力、财税、土地、工商、城镇化、社会保障、生态文明、商事制度等各项改革。

　　大力推进更深层次更高水平双向开放。着力创造更优的营商环境，深度融入“一带一路”、京津冀、环渤海、黄河金三角等区域交流合作，组织更多企业参加“山西品牌丝路行”活动，积极参加广交会、东盟博览会、亚欧博览会等国内涉外展会，着力开拓东盟、中亚、南亚等市场，扩大出口订单。更大力度建平台，推进与世界500强、央企在产业转型、园区共建等方面深化合作。加快侯马综合保税区建设，积极发展市场采购贸易、跨境电子商务和服务贸易。

　　(五)坚定不移推进农业农村改革发展。始终坚持“三农”重中之重地位不动摇，以农业供给侧结构性改革为主线，以优化产品供给、农业提质增效、农民持续增收、农村环境改善为目标，加快农业转型升级，不断巩固农业农村经济向好形势，推动农村全面小康建设迈出更大步伐。

　　全面深化农村改革，激发农村发展活力。全面完成农村土地确权工作，加快建立全市数据信息汇交、产权信息平台。落实农村承包土地“三权分置”政策，引导农民土地流转，促进土地向专业大户、农民合作社、家庭农场集中，发展多种形式的适度规模经营。鼓励返乡下乡人员到农村创业创新。加强农村集体“三资”管理，切实做好规范化建设工作。全面开展农村集体资产清产核资，积极开展农村集体产权制度改革试点工作，分类推进农村集体资产确权到户和股份合作制改革。加快推进国有林场、林下经济试点、水权制度、小型水利工程产权和农业水价等改革工作。健全农业保险制度。

　　优化农业布局，加快农产品供给结构升级。在稳粮食的前提下，大力发展特色产业，推进西山以水果、干果、杂粮为主，东山以干果、中药材、杂粮为主，沿汾河平川县(市、区)以粮食、蔬菜、水果为主，以及以尧都、侯马为中心的都市休闲农业等四大特色农业板块建设。全面推进农业标准化和品牌化创建，打造一批跨县域优质农产品品牌。力争到年底农业类山西省品牌产品达到2个，绿色产品达到5个，地理标志产品达到20个，无公害认证面积达到140万亩。稳健发展畜牧业，在基本稳定牛、羊、鸡养殖量的基础上，大力发展养猪产业，促进隰县、蒲县、大宁、乡宁等百万头生猪养殖产业化项目落地建设。实施龙头企业转型升级计划，支持农产品加工骨干企业扩大生产规模，延伸产业链条。全年农产品加工销售收入完成75亿元以上。大力发展新产业新业态，把农业生产与加工流通和休闲旅游融合，培育壮大农村新产业新业态。重点实施“221”工程，在全市认定20个市级休闲农业采摘体验园、20个乡村“农家乐”示范点、10个省级休闲农业与乡村旅游示范点，推进农村一二三产融合发展。培育壮大多种新型经营主体，促进农业加工企业转型升级。

　　推进美丽村镇建设。持续实施完善提质、农民安居、环境整治、宜居示范“四大工程”，全面改善农村条件。加大新农村建设力度，打造一批家园美、田园美、生态美、生活美的美丽乡村，创建美丽宜居示范村50个以上。

　　(六)坚定不移推进精准扶贫精准脱贫。在全力实施省八大工程二十个专项行动和市十大扶贫工程的基础上，突出抓好五方面重点工程。

　　全力实施易地扶贫搬迁“头号工程”。全面落实国家、省关于易地扶贫搬迁的新政策、新要求，把易地扶贫搬迁与发展产业、就业创业相结合，完成易地扶贫搬迁19897人，其中建档立卡贫困人口15651人，同步搬迁人口4246人。

　　着力推进特色产业扶贫“重点工程”。认真做好扶贫产业与贫困户、贫困人口对接，扶持发展杂粮、水果、蔬菜、中药材、畜牧、农产品加工和休闲观光农业等七大特色农业产业，带动10320贫困户、29656贫困人口实现脱贫。

　　大力抓好生态扶贫“试点工程”。稳步推进“五个一批”林业脱贫工作，造林绿化脱贫一批，退耕还林脱贫一批，森林管护脱贫一批，提质增效脱贫一批，林业产业脱贫一批，实现以生态建设统领脱贫攻坚，用脱贫攻坚推动生态建设，达到生态效益、经济效益、社会效益共赢。

　　引深光伏扶贫“亮点工程”。充分利用国家新能源补贴政策，在做好2016年光伏扶贫项目扫尾工作的同时，新建200座村级光伏电站，优先使乡宁、安泽、吉县3个县所有贫困村实现全覆盖，确保贫困村集体经济破零和贫困户脱贫。

　　继续开展消费扶贫“创新工程”。在现有20家消费扶贫试点企业的基础上，让更多的扶贫龙头企业加入到消费扶贫队伍中，更好地宣传推销我市贫困地区的优质产品。推进“一司一县”结对帮扶，力争使一些管理规范、经济实力强的扶贫龙头企业能够在新三板上市。

　　各位代表，小康不小康，关键看老乡。我们要以更大的决心、更精准的举措、超常规的力度，同心同德，众志成城，决战决胜脱贫攻坚。全年实现吉县、乡宁、安泽3个贫困县摘帽，204个贫困村退出、7.4万贫困人口脱贫，侯马现有贫困人口全部脱贫。

　　(七)坚定不移推进铁腕抗污治霾。环境污染是发展之患、民心之痛，环境保护是我市当前最为紧迫的一项工作。我们要以壮士断腕的决心，以更高更严的标准和要求，落实好我市《牢固树立新发展理念全面加强生态环境保护的意见》，实施好我市《环保突出问题暨环保部约谈问题整改工作实施方案》，扎实推进科学治污、精准治污、依法治污、综合治污、联动治污、全民治污、转型治污，坚决打好打赢环境治理攻坚战。

　　严治工业污染。坚持红线管理、提标改造、超低排放，确立临汾标准。严格落实停产限产、错峰生产、驻厂监管、常态突查等措施，对钢铁、焦化和其他涉气工业企业实施深度治理。全面整治“小散乱污”企业，市区155平方公里规划区及周边区域所有“小散乱污”企业全部关停，其他区域“小散乱污”企业分类采取关停、整合、升级、改造或转产等措施，最大程度削减工业排放量。

　　狠抓燃煤污染。对市区155平方公里规划区内所有燃煤锅炉实行改造、置换，3月底前全部淘汰“清零”，今年新增集中供热面积389万平方米，两年内新增清洁供热面积793万平方米，实现“无煤化”。对市区周边1500平方公里区域内10t/h及以下燃煤锅炉和全市所有行政事业单位营业性锅炉、茶浴炉全部进行“煤改电”“煤改气”清洁能源改造，4月底前全部完成，实现“清洁化”。对1500平方公里区域外平川县市区所有燃煤锅炉实施深度治理，达到特别排放限值标准;非平川区域燃煤锅炉全部升级污染治理设施，达到新建锅炉污染物排放标准，实现“集约化”。

　　整治面源污染。强化扬尘治理，严格落实建筑工地施工围挡、物料覆盖、车辆冲洗、地面硬化、洒水抑尘5个100%措施，加大市区道路及交通干线保洁频次。强化餐饮油烟治理，持续推进市区夜市烧烤、露天摊点专项整治，市区所有产生油烟餐饮行业加装油烟净化装置，达到排放标准。强化机动车污染防治，持续推进老旧车辆淘汰，减少尾气排放污染。市区逐步推广纯电动出租车，平川县市基本实现纯电动公交车全覆盖。市区新投放“共享单车”1万辆，平川县市公共自行车力争全覆盖。

　　推进水土防治。制定水污染防治2017年行动计划，做好饮用水水源地规范化建设和不达标饮用水治理，确保全市集中式饮用水源地供水水质达标率100%。全面推行“河长制”，开展重点河流生态环境综合整治。完成浍河、昕水河等重点河流人工湿地建设，稳步提升地表水水质。划定畜禽养殖禁养区，完成农村生活污水综合利用示范项目建设。推进焦化废水、煤矿矿井水深度治理。认真落实《土十条》，编制并实施市级及各县(市、区)土壤污染防治行动计划。完成全市土壤污染风险管控调查与评估。开展土壤污染防治试点工作。

　　加强环境监管。完善属地管理、分级负责四级网格监管体系。建立全市大气污染防治监控大数据平台，采取无人机、视频在线、移动走航车等方式，加强监测监控。上收全市环境空气质量监测事权。全力推进地方环保立法，制定《临汾市区燃煤污染防治规定》和《临汾市禁止燃放烟花爆竹安全管理规定》两部地方性法规，为整治燃煤污染和管控二氧化硫提供法律保障。推动环保执法与刑事司法联动、人大执法联动。

　　提升环保基础。加强污染源解析工作;加快提标改造和配套污水收集管网建设，全市生活污水收集率达到75%以上;加快市第二污水处理厂管网建设，稳步提升负荷率，完成建设市第三污水处理厂;市区和各县(市、区)城市生活垃圾处理场全部建成投运;建立农村生活垃圾处理运行管理长效机制，实现农村垃圾规范收集、合理处置;推进全市工业园区污水集中处理。

　　加快生态建设。大力开展造林绿化，完成营造林49.14万亩，全市森林覆盖率提高1个百分点以上。加大小流域治理力度，加快推进汾河、涝洰河等重点流域生态修复项目。持续推进西山七县主体功能区建设，实施采煤沉陷区、水土流失区、煤矸石山等环境治理修复工程。

　　各位代表，“子规夜半犹啼血，不信东风唤不回”。在这场环保攻坚战中，复杂性和艰巨性我们是有深刻估计和思想准备的，但是我们别无退路，别无选择，只能义无反顾，勇往直前，坚持不懈地推进标本兼治，坚持不懈地抓好源头治理，让临汾天更蓝、山更绿、水更清、空气更清新!

　　(八)坚定不移推进城乡协调发展。

　　加快中心城市建设。围绕“建设新城、改造老城、提升东城、绿色绕城”的思路，实施新建道路、桥梁枢纽、街巷改造、园林绿化、环城水系、市政管线、污废处置、信息网络、社会公益和保障安居等十大类170项城建项目，当年完成投资173.3亿元。重点新建规划三街、机场快速通道等29条道路，加快打通“断头路”，改造兵站路、科委巷等14条街巷，建成滨河西路与彩虹桥景观大道立交桥、迎宾大道与滨河东路立交桥等6座桥梁，实施漪汾花园公租房、北城濠西段棚户区改造、高河店城中村改造等30项保障安居项目。对人口密度过大的老城区原则上“只拆不建”，腾出空间用于绿地、停车场和基础设施配套。创建“公交都市”，建设“掌上公交”，打造“智慧交通”。

　　优化城镇空间布局和功能。按照“沿汾崛起、两翼齐飞”的总体布局和“一带两圈多点”城镇化布局，有序调整行政区划，加快“一城三区”、侯马曲沃同城化发展;继续实施“大县城战略”，积极推进产城融合，建设一批特色小城镇，加快构建多极发展新格局。

　　统筹城乡基础设施建设。加强城市综合交通网络、地下综合管廊建设。完善城乡道路交通体系，完成公路建设投资50亿元，其中续建高速公路148公里，完成农村公路850公里。积极推进城市供水、燃气、供热、污水和垃圾处理向周边村镇延伸。实施农村基础设施提升工程。

　　完善一体化推进机制。加快推进规划编制、基础设施、公共服务、产业布局、制度安排“五个一体化”。探索实施城镇建设用地增加规模同吸纳农业转移人口落户数量挂钩机制。鼓励就近城镇化、梯度城镇化，出台财政支持政策，促进符合条件的农业转移人口有序落户城镇。

　　(九)坚定不移推进安全稳定工作。牢固树立“总体安全观”，以铁的担当尽责，铁的手腕治患，铁的心肠问责，铁的办法治本，强化安全监管，推进社会治理创新，为全市经济发展提供可靠的安全保障。

　　全面压实安全责任。严格落实“党政同责、一岗双责、尽职履责、失职追责”要求，将安全生产目标责任逐级分解，进一步明确各级党委、政府领导责任和部门监管责任。严格落实企业主体责任，切实做到安全责任、管理、投入、培训和应急救援“五到位”。

　　坚持依法依规治安。坚决贯彻落实中央《关于推进安全生产领域改革发展的意见》和新修订的《山西省安全生产条例》，进一步健全安全联合执法工作机制，对违法违规行为零容忍、严查处。对典型事故和瞒报、谎报、迟报行为，一律提级调查。

　　深入整治安全隐患。坚持“四不两直”突查和“双随机”检查方式，全面开展安全隐患大排查大整治，切实抓好煤矿、非煤矿山、道路交通、危化品、地质灾害、森林防火、食品药品等重点行业领域安全生产工作，及时消除事故隐患。对重大隐患实行挂牌督办、限期整改，对非法生产经营建设行为坚决予以打击取缔。加大安全生产考核权重，严格落实“一票否决”，确保全市安全生产形势持续稳定好转。

　　全力维护社会稳定。落实信访工作责任制和重点案件领导包案制度，建立畅通、规范、有序的群众诉求表达渠道。积极推动社会治理创新，深入推进“平安临汾”“法治临汾”建设，加快建设立体化、信息化社会治安防控体系，切实提高群众的安全感和满意度。强化互联网管理，健全网上网下一体化打防管控机制。强化应急救援管理，积极推进应急处置信息共享机制，妥善处理各类突发事件，确保社会和谐稳定。

　　(十)坚定不移推进民生改善。为政之要，民生至上。我们要顺应人民群众共建共享的美好愿景，致力于增进民生福祉，让发展更有“温度”、幸福更有“质感”。无论今年财政压力有多大，用于民生的支出只增不降，为群众办的实事只加不减。

　　大力加强文化建设。充分发挥文化对丰富群众精神生活、构建核心价值、共创精神家园的作用，重点推动市图书馆、奥体中心体育场、文化中心等标志性文化建筑和县级“四馆一场一院”建设，夯实文化阵地。促进全民阅读、全民健身。

　　优先发展教育事业。大力实施学前教育奠基、义务教育均衡、普通高中教育提升、职业教育接轨、“三优”培养选拔等五大工程，提升教育教学质量，办好人民满意的教育。全力推进山西师大和师大临汾学院新校区建设。

　　切实加强卫计事业。全面推进卫生与健康改革发展，加快“健康临汾”建设，深化医药卫生体制改革，重点启动城市公立医院综合改革。积极稳妥实施全面两孩政策。大力开展爱国卫生运动，加强重大疾病防控，推进基本公共卫生计生服务均等化，努力提升健康服务水平。大力推动中医药创新。

　　千方百计扩大就业。扎实开展就业援助专项行动，全面做好高校毕业生、农村转移劳动力、城镇失业人员、退役军人等群体就业工作。加强职业培训，提升劳动者就业创业和职业转换能力。

　　织牢社会保障安全网。健全社会救助体系，完善社会救助标准与物价上涨联动机制，兜住困难群众民生底线。推动机关事业单位养老保险制度改革入轨运行。在全市建立统一的城乡居民基本医保制度。适时推进长期护理保险制度。推进和完善养老服务体系建设，不断满足养老服务多样化需求。继续加大各类保障房建设力度。加快社会公共法律服务体系建设。

　　在做好上述工作的同时，还要加快推进国家信息惠民城市试点建设，着力落实城市云中心重点工程项目及各项信息惠民应用系统建设;深入开展“双拥共建”活动，做好国防动员、人民防空、民兵预备役工作;支持工会、妇联、共青团、工商联等人民团体工作;统筹审计、统计、工商、质监、食药监、集体经济、档案、气象、水文、地震、地方志、民族宗教、外事侨务、对台、老龄和残疾人等各项工作;继续做好对口援疆、第三次全国农业普查工作。

　　各位代表，人民群众对美好生活的向往就是我们的奋斗目标。我们要牢固树立以人民为中心的发展思想，在继续改善人民群众生产生活条件的基础上，更加注重提高人的素质，促进人的全面发展。今年，市政府继续办好6件民生实事：一是实施学前教育优质普惠工程。按照公办与民办并重、城市与农村并重、建设与规范并重的原则，在市区、城区、中心城镇建成公办中心幼儿园、骨干幼儿园覆盖网络，在农村义务教育学校建成幼儿教学班覆盖网络，解决入园难、入园贵问题。二是实施乡村医疗服务提质工程。健全药品供应保障机制，加强药品价格管理。推进建立分级诊疗制度。落实乡村两级医务人员、预防保健人员工资待遇，健全防治网络。三是实施农业强基工程。改善农业基础条件，完成高标准农田建设3万亩以上;全力推动县域小水网工程。四是实施饮水安全工程。做好龙祠等水源地保护工作，加强水质检测，抓好农村饮水安全巩固提升工程建设。五是实施城市综合整治工程。以整治“脏、乱、差、堵”为重点，积极开展市容环境专项整治、扬尘污染专项治理、“三供两处理”运营考核等工作，不断提升城市管理工作水平。六是实施就业创业援助工程。落实省政府扶持高校毕业生创业就业“七补一贷”政策，推进高校毕业生创业园区建设。实施职业培训全覆盖计划和新生代农民工职业技能提升计划，新转移农村劳动力4.7万人。

　　三、全面提升政府自身建设水平

　　新形势、新任务对政府工作提出了更高要求。我们要牢固树立“四个意识”特别是核心意识、看齐意识，更加坚定地维护以习近平同志为核心的党中央权威，更加自觉地在思想上政治上行动上同党中央保持高度一致，以实际行动落实好党中央的各项决策部署。强化宗旨意识，忠实履行职责，与中央要求“对表”、与发达地区对标、与国际通行惯例对接，加强人民政府、法治政府、效能政府、担当政府、廉洁政府建设，不断提升政府治理体系和治理能力现代化水平。

　　提升干部队伍素质。持续举办政府系统领导干部能力提升系列讲座，加强干部政治理论、金融、法律、科技、互联网等方面的学习培训，优化政府工作人员知识结构、能力结构。完善政府领导集体学习制度、专家授课制度，健全公务员培训制度，推进政府“智库”建设，提升科学决策能力和水平。

　　锤炼务实为民作风。深入开展“讲政治、知敬畏、守规矩”专项治理活动，严厉查处有令不行、有禁不止和不作为、慢作为、乱作为行为，把担当精神转化为推动发展的动力。加大政务公开力度，全面推进行政决策、执行、管理、服务、结果公开，保障人民群众的知情权、参与权、表达权、监督权。创新联系群众方法，完善“市长信箱”“市长热线”“临汾随手拍”等平台，加强政府与群众的沟通互动。借助互联网、新媒体，开展问政和评议活动，多渠道了解社情民意。

　　强化民主法治理念。加强法治政府建设，严格遵循重大决策公众参与、专家论证、风险评估、廉洁性审查、合法性审查和集体讨论决定等法定程序，提高工作法治化、科学化、民主化水平。率先建立法律顾问和公职律师制度。主动接受人大及其常委会法律和工作监督，接受政协民主监督，认真办理人大代表议案建议、政协委员提案。高度重视社会舆论监督，把压力变成为群众谋利益的动力。深化综合行政执法体制改革，重点做好市场监管、卫生计生、文化旅游、商务流通、城乡建设、城市管理等领域改革工作，解决多头执法和多层次执法问题。全面推开“双随机、一公开”监管，强化线上线下一体化监管。

　　提高行政办事效率。加强市政务服务中心建设，完善全市一张网政务服务平台，让企业和群众办事更方便。深入落实“13710”工作制度，6月底前市、县、乡全面建立电子督办平台，将重点工程项目、企业技改项目和招商引资项目纳入平台管理，切实提高工作效率，推动政府各项工作见人见事、见根见果，抓细抓小、抓紧抓实。加快推行政府系统OA移动办公，健全完善电视电话会议、网络视频会议等系统，合理控制会议数量与规模。

　　坚持廉洁自律操守。深入推进全面从严治党，严格规范党内政治生活，严守政治纪律和政治规矩，运用好监督执纪“四种形态”，认真落实主体责任和“一岗双责”，做到“两手硬、两促进”。推动中央八项规定精神风化俗成，坚决防止“四风”反弹回潮，带动政风民风进一步好转。开展环保领域、扶贫领域专项整治，对履职不到位、监管不严、弄虚作假的要坚决问责追责，对向环保资金、扶贫款物伸手的要坚决查处。支持监察体制改革，为改革提供必要的人、财、物等保障。加大审计监督力度，推进公共资金、国有资产、国有资源、领导干部经济责任审计全覆盖。坚持用制度管权、管事、管人，加强责任追究的制度化、程序化和常态化建设，从源头上预防腐败。

　　各位代表!九层之台，起于累土。大道至简，实干为要。让我们在市委的坚强领导下，在市人大、市政协和社会各界的监督支持下，撸起袖子加油干，扑下身子抓落实，一锤接着一锤敲，一步一个脚印走，凝心聚力，攻坚克难，为决胜全面小康，实现振兴崛起，建设富裕、文明、绿色、幸福新临汾而不懈努力，以优异成绩迎接党的十九大胜利召开!
